# PSMC2 promotes the progression of gastric cancer via induction of RPS15A/mTOR pathway

**DOI:** 10.1038/s41389-022-00386-7

**Published:** 2022-03-07

**Authors:** Tao Liu, Junling Zhang, Hu Chen, Tashi Bianba, Yisheng Pan, Xin Wang, Yong Jiang, Zhen Yang

**Affiliations:** 1 Gastrointestinal Surgery, the first affiliated hospital of Zhengzhou University, Zhengzhou, China; 2 Department of General Surgery, Peking University First Hospital, No.8 XiShiKu St., Xicheng District, Beijing, China; 3 Department of General Surgery, Tibet Autonomous Region People’s Hospital, No.18 Linkor North Roud., Cheng Guan District, Lasha, Tibet Autonomous Region China

**Keywords:** Gastric cancer, Cell biology, Molecular biology

## Abstract

As one of the most common malignant tumors, it is particularly important to further understand the development mechanism of gastric cancer and to find more effective therapeutic target genes. The results of immunohistochemical staining showed that PSMC2 was upregulated in gastric cancer. Cell function experiments indicated that PSMC2 knockdown inhibited the proliferation, clone formation and migration of gastric cancer cells, and induced apoptosis. In vivo experiments further showed that PSMC2 knockdown suppressed tumor growth. RPS15A and mTOR pathway were identified the downstream gene and pathway of PSMC2 by GeneChip and IPA. PSMC2 knockdown inhibited RPS15A expression and mTOR pathway, which was neutralized by RPS15A overexpression. Overexpression of RPS15A promoted the proliferation and migration of gastric cancer cells, which alleviated the inhibitory effect caused by PSMC2 knockdown to a certain extent. The mTOR pathway inhibitor Torin1 partially restored the promoting role of RPS15A overexpression on the gastric cancer cell proliferation. Furthermore, bioinformatics analysis and dual-luciferase reporter assays showed that PSMC2 and RPS15A competitively bound to hsa-let-7c-3p. Inhibition of hsa-let-7c-3p promoted the migration of MGC-803 cells and reduced the apoptosis level, while simultaneous inhibition PSMC2 and hsa-let-7c-3p restored the migration and apoptosis levels of gastric cancer cells. In conclusion, PSMC2 and RPS15A were highly expressed in gastric cancer. PSMC2 enhanced RPS15A levels by targeting hsa-let-7c-3p, and then activated mTOR pathway, thereby promoting the progression of gastric cancer.
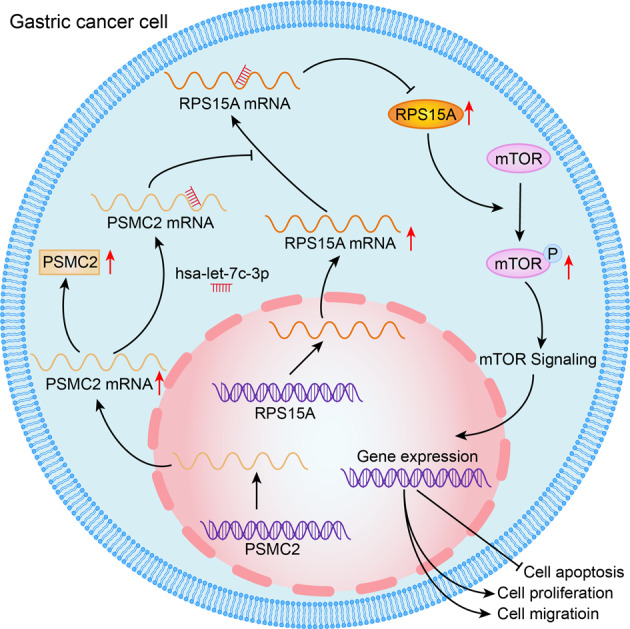

## Introduction

Gastric cancer is one of the most common malignant tumors. More than 1 million new cases of gastric cancer have been reported in 2020, and the incidence rate ranks fifth in global. The incidence rate is twice as high as in men as in women, and is higher in South Central and East Asian counties. Gastric cancer also has a high mortality, ranking fourth in the world, with an estimated 769,000 deaths from gastric cancer in 2020 [1]. Helicobacter pylori is a major cause of gastric cancer, and other risk factors include smoking, alcohol consumption, familial susceptibility, a history of gastric surgery, and so on [2]. Gastric cancer is a heterogeneous group of diseases, including 3 different subtypes: distal intestinal-type gastric cancer with chronic gastritis and Helicobacter pylori infection, proximal intestinal-type gastroesophageal cancers, diffuse signet-ring cell type cancers [3]. At present, surgery or endoscopic resection is an important method for the treatment of gastric cancer. Early gastric cancer is often asymptomatic, leading to delayed diagnosis and missed the opportunity of radical surgery [4]. Although the use of adjuvant chemotherapy and chemoradiotherapy improves the survival rate of patients with advanced gastric cancer and improves the survival and quality of life of patients with advanced or metastatic gastric cancer [1, 2]. However, the 5-year overall survival rates of patients with local and distant metastasis of gastric cancer are only 30% and 5%, respectively [5]. Therefore, it is particularly important to further understand the development mechanism of gastric cancer and find more effective therapeutic target genes.

26S proteasome plays a vital role in the selective degradation of eukaryotic proteins by recognizing ubiquitinated substrates, and then participates in many cellular processes, such as cell cycle, apoptosis, immune response, verification and response to protein toxic stress [6]. One or two 19S regulatory complexes combine with a single 20S catalytic complex to form a single- or double-capped (26S1 or 26S2) intact 26S proteasome, respectively [7]. Proteasome 26S subunit ATPase 2 (PSMC2) is a part of the 19S regulatory complex, which catalyzes the unfolding and transport of substrates into the 20S proteasome [8]. PSMC2 expression is crucial for the assembly of 19S and 26S proteasome [9]. As a CYCLOPS (Copy-number alterations Yielding Cancer Liabilities Owing to Partial losS) gene, PSMC2 is excessively expressed in normal cells, which protects cell from PSMC2 inhibition by forming a complex, and is associated with cell proliferation or survival [10]. It has been reported that PSMC2 is highly expressed in the postmenopausal osteoporosis model of ovariectomized (OVX) mice. Knockdown of PSMC2 promotes the osteogenic differentiation of bone marrow mesenchymal stem cells in vitro, promotes bone formation, and improves the biological characteristics of the femur of OVX mice [11]. Song *et al*. determined the high protein level of PSMC2 in samples from osteosarcoma, and used in vivo and in vitro experiments to demonstrated the inhibitory effect of PSMC2 silencing on osteosarcoma [12]. Su et al. found that PSMC2 had high diagnostic value for HPV-negative head and neck squamous cell carcinoma and adjacent tissues [13]. Besides, PSMC2 is highly expressed in kidney cancer cells and has a tumor-promoting effect on kidney cancer [14]. Therefore, PSMC2 is also considered to be an important factor in tumor occurrence and development. However, the functional verification and mechanism research inhibition of PSMC2 in the progression of gastric cancer are lacking.

In this study, we found that PSMC2 was significantly highly expressed in gastric cancer tissues, and its expression in patients with gastric cancer was correlated with tumor characteristic such as pathological stage, T Infiltrate, the expression of Ki67 and age. We will focus on the function and mechanism of PSMC2 in the progression of gastric cancer, and further verify it at the cellular and animal levels.

## Materials and methods

### Tissue chip and cell culture

The tissue chip was purchased from Shanghai Outdo Biotech Company (XT15-042). There were 74 cases of para-carcinoma tissues and 93 cases of gastric cancer tissues. All patients have signed informed consent. The related experiments involved in this study have also been approved by the Ethics Committee of the Peking University First Hospital.

The gastric cancer cells AGS, SGC-7901, and MGC-803 used in this study were purchased from BeNa. AGS cells were cultured in F12 medium with 10% FBS (16000-044, Invitrogen). SGC-7901 and MGC-803 cells were cultured in RPMI 1640 medium (10-040-CVB, corning) with 10% FBS. All cells were cultured in 37°C incubator containing 5% CO_2_. Torin1 (HY-13003), the inhibitor of mTOR pathway, was purchased form MedChrmExpress (MCE, China), and 60 μM Torin1 was added to the cell culture medium 24 h after the cells were infected with lentivirus.

### Immuohistochemical staining

Gastric cancer and para-carcinoma tissues were made into 5 μm-thick paraffin sections. After dewaxing, the sections were subjected to antigen repaired with citrate (Maxim Biotechnologies). The sections were blocked in 3% H_2_O_2_ for 5 min and then in 5% serum for 15 min. The diluted primary antibody solutions were incubated with sections at 37 °C for 1 h. The sections were washed three times with 1 X PBST buffer, 5 min each time. The diluted secondary antibody solutions were incubated with sections at 37 °C for 1 h. The sections were washed with 1 X PBST buffer for 5 min three times. DBA dye solution was used to stain the sections for 5 min in the dark. After washing, sections were redyed with hematoxylin (Baso) for 10 s. Finally, the sections were dehydrated, transparent and sealed. The results were judged by the proportion of positive cells and staining intensity under microscope. The primary and secondary antibodies involved in this experiment were provided in Supplementary Table 1.

### Gene knockdown or overexpression gastric cancer cell model

Using PSMC2 or RPS15A gene as template, three corresponding RNA interference target sequences were designed respectively, and synthesized single-stranded DNA oligo containing interference sequences. The single-stranded DNA oligo was annealed to form double-stranded DNA, which was connected with the lentiviral vector through the restriction sites to construct the target gene RNA interference lentiviral vectors. The primer amplification sequence was designed by a PSMC2 or RPS15A basis, and then recombined with the linearized carrier in vitro. The recombinant plasmid was attached to the lentiviral vector to construct the target gene overexpression lentiviral vectors. The lentiviral vectors were labeled with green fluorescent protein (GFP). Gastric cancer cells (1 ×10^7^ cells/well) were infected with 1 ×10^7^ TU/ml shPSMC2 lentivirus or 2 ×10^8^ TU/ml shRPS15A lentivirus. After 18 h, the new medium was replaced and then cultured for 72 h. Fluorescence was observed under a microscope (IX71, Olympus) to evaluate the efficiency of cell infection. Target sequences and shRNA sequences used for gene knockdown were provided in Supplementary Table 2.

### Real-time Quantitative PCR Detecting System (qPCR)

Gastric cancer cells were collected and lysed with 1 ml Trizol. The total RNA was extracted according to Trizol (T9424-100m) operating instructions of Sigma company. 1.0 μg of total RNA was subjected reverse transcription using Hiscript QRT supermax for qPCR (+gDNA WIPER) (R123-01, Vazyme) under the guidance of the operating instructions to obtain cDNA. cDNA, primers and SYBR Green master max (Q111-02, Vazyme) were configured in a certain proportion of the reaction system, and a two-step Real-time PCR (VII7, ABI) was performed. The calculation formula of gene relative quantitative analysis results: F = 2^−△△Ct^. GAPDH was the internal reference. This experiment was repeated 3 times independently. Primers used in qPCR were provided in Supplementary Table 3.

### Western blotting (WB)

The gastric cancer cells were lysed using 1 X lysis buffer and the total protein was extracted. BCA Protein Assay Kit (23225, HyClone-Pierce) was used to detect protein concentration. 20 μg of protein was taken for sodium dodecyl sulfate polyacrylamide gel electrophoresis (SDS-PAGE), and then transferred to Polyvinylidene Fluoride (PVDF) membranes. PVDF membranes were blocked in 1 X TBST solution containing 5% skimmed milk for 1 h at room temperature, and incubated with the diluted primary antibodies for 2 h at room temperature. 1 X TBST solution was employed to wash the membranes 3 times, 10 min each time. PVDF membranes were incubated in dilute secondary antibodies for 1 h at room temperature. After washing membranes 3 times, the chemiluminescence was carried out by using the immobilon Western chemiluminescent HRP Substrote Kit (RPN2232, Millipore), and the chemiluminescence imaging system (AI600, GE) was used for imaging. GAPDH was used as an internal reference. The primary and secondary antibodies involved in this experiment were provided in Supplementary Table 1.

### MTT assay

AGS and MGC-803 cells in the logarithmic growth phase were seeded into 96-well plates with 2000 cells per well. The OD value of cell solution was detected on the 1st, 2nd, 3rd, 4th and 5th day after transfer to 96-well plates. 20 μl 5 mg/ml MTT solution (JT343, Genview) was added to each well, and incubated for 4 h. Then the culture medium was discarded, and 100 μl DMSO solution (130701, Shanghai Shiyi Chemical Reagent Co., Ltd.) was added into each well, and incubated for 5 min. The OD value of the cell solution at the wavelength of 490/570 nm was detected by microplate reader (M2009PR, Tecan infinite). This assay was performed 3 independent repetitions.

### Clone formation assay

AGS and MGC-803 cells infected with shRNA or shPSMC2 lentivirus were transferred to 6-well plate with 500 cells per well, and cultured for 8 days. The cells were washed once with PBS, and then 1 ml of 4% polyformaldehyde (Sinopharm Chemical Reagent Co., Ltd) was added to fix the cells for 30–60 min. The cells were washed once with PBS, and the GIEMSA dye (AR-0752, Shanghai Dingguo Biotechnology Co. Ltd) was added to each well for staining for 15 min. Cells were washed with ddH_2_O, and taken pictures with a with digital cameras (DSC-HX300, SONY) after drying. The number of clones was counted (each clone contains more than 50 cells). There were 3 independent repetitions in this assay.

### Flow Cytometer (FCM)

The apoptosis of gastric cancer cells (AGS and MGC-803) was detected by Annexin V-APC single staining flow cytometry. When the coverage of gastric cancer cells infected with lentivirus reached 85%, the cells were collected after digestion with trypsin. After washing the cells with D-Hanks pre-cooled at 4 °C, cells were washed again with 1 X binding buffer. The cells were resuspended into 200 μl 1 X binding buffer, and stained with 10 μl Annexin V-APC (88-8007-74, ebioscience) at room temperature and dark for 10–15 min. Flow cytometry (Guava easyCyte HT, Millipore) was used to analyze cell apoptosis. This assay was repeated 3 times.

### Transwell assay

Transwell chambers (3422, Corning) were placed in empty 24-well plates, each chamber was added with 100 μl serum-free medium, and placed in 37 °C incubator for 1–2 h. After removing the chamber medium, 100 μl cell suspension (containing 60000 cells) was added into each chamber, and 600 μl medium containing 30% FBS was added into the lower chamber. The chambers were transferred into the lower chambers containing 30% FBS medium and cultured in 37°C incubator for 30 h. The culture medium in the chambers was removed and the non-metastatic cells were removed with a cotton swab. 400 μl GIESMA dye was added to the empty 24-well plate, and the chambers were soaked for 5 min to stain the migrating cells. After washing, the cells were photographed under microscope and the migrating cells were counted. This assay was carried out 3 independent repetitions.

### Wound healing assay

Gastric cancer cells infected with lentivirus were inoculated into 96-well plates with 50000 cells per well. The next day, the medium was replaced with a medium containing 0.5% FBS. The 96 Wounding Replicator (VP408FH, VP scientific) was used to push out a scratch in the middle of the 96-well plate. 96-well plates were washed with serum-free medium for three times. The cells were cultured in 0.5% FBS medium and photographed at 0 h, 24 h and 48 h after scratch formation using a fluorescence microscope (IX73, OLYMPUS). According to the pictures after scratch, the cell migration rate of each group was calculated. This assay was repeated 3 times independently.

### Human Apoptosis Antibody Array

Human Apoptosis Antibody Array (ab134001) was purchased from abcam to evaluate the effects of PSMC2 knockdown on the expression of proteins in Human apoptosis pathway. SGC-7901 cells infected with shPSMC2 or shCtrl lentivirus were lysed with 1 X cell lysis buffer and the total protein was extracted. The total protein concentration of each lysate was determined by BCA Protein Assay Kit. The members were incubated with 1 X blocking buffer at room temperature for 30 min with gentle rocking, and then incubated with 1.2 ml of samples overnight at 4 °C with gentle rocking. After washing 3 time, the members were incubated with 1 ml of 1 X biotin-conjugated anti-cytokines overnight at 4 °C with gentle rocking, and washed 3 times, 5 min each time. The members were incubated with 1.5 ml of 1 X streptavidin-HRP at room temperature for 2 h. A chemiluminescence imaging system was used to detect the signal of the members. This assay was repeated 2 times.

### Subcutaneous injection tumor model

4-week-old female BALB/c nude mice were purchased from Shanghai Lingchang Biotechnology Co., Ltd. to construct a tumor nude mouse model by subcutaneous injection, and 10 nude mice were randomly assigned to each group. All animal experiments were approved by the Ethics Committee of Laboratory Animal Care and Use of Peking University First Hospital (Approval No. J2020-14). All animals were treated according to the standards prescribed by the “Guide-lines for the welfare and use of animals in cancer research”. MGC-803 cells (4.0 ×10^6^ cells) infected with shPSMC2 and negative control shRNA lentivirus were injected subcutaneously into the right forelimb armpit of nude mice. The tumor volume and mouse weight were measured 13 days after injection to draw the tumor growth curve. After 22 days of injection, nude mice were anesthetized by intraperitoneal injection of 0.7% Pentobarbital Sodium at 10 μl/g dose. Then nude mice were placed on an in vivo imaging instrument (LB983, Berthold Techologies) for imaging, and the fluorescence intensity was observed. After the nude mice were sacrificed, the tumors were taken out from the nude mice and photographed with a digital camera (DSC-HX300, SONY). The tumor volume and weight were measured. Immunohistochemical staining was performed in accordance with the method described above to evaluate the expression of Ki67 in tumor tissues.

### GeneChip and Ingenuity pathway analysis (IPA)

Total RNA was extracted from SGC-7901 cells infected with shPSMC2 or shCtrl lentivirus and quantified. Under the guidance of the operation instructions, the whole gene expression profile on GeneChip primeview was analyzed. After preprocessing the chip data such as data normalization and data cleaning, hierarchical clustering difference analysis was performed by using Limma package in R studio, and differentially expressed genes were screened using |logFold Change| ≥ 1.3 and FDR < 0.05 as the criteria. IPA software was used to construct the interaction network of PSMC2, PSMC2 downstream genes and PSMC2 related signaling pathways.

### CCK8 assay

AGS and MGC-803 cells infected lentivirus in the logarithmic growth phase were resuspended after tryptic digestion. 100 μl cell suspension was transferred to 96 well plate with a density of 2000 cells per well. The proliferation of AGS and MGC-803 cells was detected on day 1, 2, 3, 4, and 5. 10 μl CCK8 regent (96992, Sigma) was added to each well for 4 h. The 96 well plate was oscillated on the oscillator for 2 min, and then the OD value at 450 nm wavelength was measured using the microplate reader (Tecan infinite), and a 5-day cell proliferation curve was drawn. There are 3 independent repetitions in this assay.

### Dual-luciferase reporter assays

The 3’UTR of PSMC2 and RPS15A contained potential binding sites with hsa-let-7c-3p. The 3’UTR of PSMC2 and RPS15A harboring wild-type or hsa-let-7c-3p potential binding site was inserted into the psiCHECK^TM^-2 dual-luciferase reporter vector. 293T cells were inoculated to 96 well plate. Wild-type or mutant reporter plasmids were transfected with hsa-let-7c-3p or control to 293T cells using Lipofectamine 3000 (Invitrogen). After transfecting 48 h, the cells were collected, luciferase activity was detected using Promega Dual-luciferase System (Promega) and standardized with Renilla luciferase. This assay was repeated 3 times.

### Statistical analysis

All cell experiments were independently repeated 3 times. All data in this study were expressed as mean ± standard deviation (SD). SPSS was used for statistical analysis, and GraphPad was used to draw statistical graphs. Sign test was used to analyze the expression of PSMC2 gene in gastric cancer tissues and para-carcinoma tissues. Mann Whitney *U* was used to analyze the expression differences of PSMC2 gene in tumor characteristic. Spearman correlation analysis (two-tailed) was used to analyze the correlation between PSMC2 gene expression and pathological stage, Tumor Infiltrate, the expression of Ki67 and age. T-test was used for statistical analysis between the two groups. *P* < 0.05 indicated that the difference was statistically significant.

## Results

### PSMC2 was highly expressed in gastric cancer

**Fig. 1:**
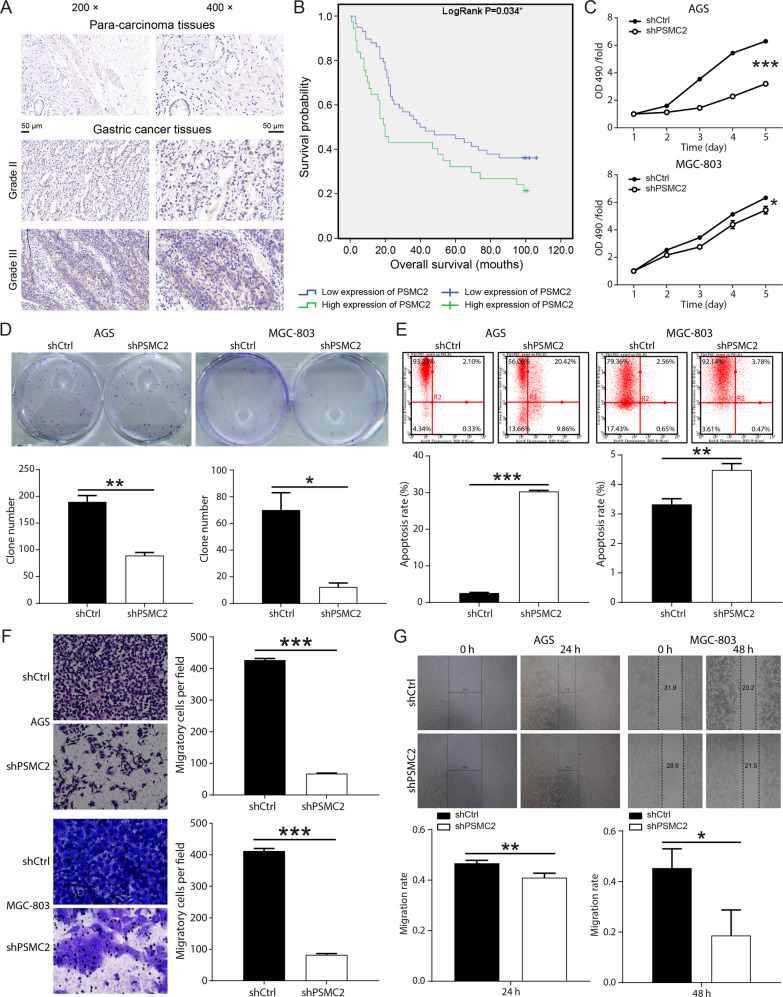
PSMC2 was upregulated in gastric cancer and the role of PSMC2 in gastric cancer cells was studied. (AGS and MGC-803 cells). **A** Immumohistochemical staining was conducted to evaluate the expression of PSMC2 in the gastric cancer tissues and para-carcinoma tissues. **B** Kaplan–Meier was performed to analyze the relationship between PSMC2 expression and overall survival rate of patients with gastric cancer. **C** The results of MTT assay showed that after PSMC2 was knocked down, the proliferation fold of gastric cancer cells was significantly reduced. **D** The results of clone formation assay indicated that downregulation of PMSC2 inhibited the clone formation ability of gastric cancer cells (AGS and MGC-803). **E** Flow cytometry was performed to evaluate the apoptosis level of gastric cancer cells infected with shPSMC2 or shCtrl lentivirus. **F** Transwell assay was conducted to determine the changes in the migration ability of gastric cancer cells after knocking down PSMC2. **G** Wound healing assay was conducted to determine the changes in the migration ability of gastric cancer cells after knocking down PSMC2. *P < 0.05; **P < 0.01; ***P < 0.001, compared with shCtrl group. Error bars meant SD.

The results of immunohistochemical staining determined that PSMC2 was highly expressed in the gastric cancer tissues (Fig. 1A). The analysis results of Sign test indicated that the expression of PSMC2 was statistically different in gastric cancer and para-carcinoma tissues (Table 1). Further statistical analysis suggested that the expression of PSMC2 in gastric cancer patients showed statistically differences in tumor characteristic such as pathological stage, Tumor Infiltrate, Ki67 expression and age (Table 2). Spearman correlation analysis determined that the expression of PSMC2 was positively correlated with pathological stage, Tumor Infiltrate, Ki67 expression and age (Table 3). According to Kaplan–Meier survival analysis results, PSMC2 expression was significantly correlated with overall survival of gastric cancer patients, that was, the high expression of PSMC2 indicated a poor prognosis (Fig. 1B). All of these results suggested that PSMC2 might play a vital role in the development and prognosis of gastric cancer.

**Table 1:** Expression patterns in gastric cancer tissues and para-carcinoma tissues revealed in immunohistochemistry analysis.

PSMC2 expression	Tumor tissue		Para-carcinoma tissue		*P* value
	Cases	Percentage	Cases	Percentage	
Low	57	61.3%	74	100%	<0.001***
High	36	38.7%	0	–%

**Table 2:** Relationship between PSMC2 expression and tumor characteristics in patients with gastric cancer.

Features	No. of patients	PSMC2 expression		*P* value
		Low	High	
All patients	93	57	36	
Age (years)				0.016*
≤65	47	35	12	
>65	44	22	22	
Gender				0.244
Male	61	40	21	
Female	32	17	15	
Grade				0.715
2	13	7	6	
3	70	44	26	
4	10	6	4	
Tumor Infiltrate				0.003**
T1	7	7	0	
T2	7	5	2	
T3	59	37	22	
T4	19	7	12	
Lymphatic metastasis (N)				0.088
N0	24	18	6	
N1	13	8	5	
N2	25	14	11	
N3	29	15	14	
Tumor metastasis				0.512
No	84	52	32	
Yes	8	4	4	
Stage				0.011*
1	9	7	2	
2	28	22	6	
3	47	22	25	
4	7	4	3	
Tumor size				0.156
<6 cm	45	31	14	
≥6 cm	46	25	21	
Lymph node positive				0.077
≤4	46	32	14	
>4	43	22	21	
Expression of Ki67				0.015*
Low	51	37	14	
High	42	20	22

**Table 3:** Relationship between PSMC2 expression and tumor characteristics in patients with gastric cancer.

		PSMC2
Stage	Pearson correlation	0.266
	Significance (two-tailed)	0.011*
	*N*	91
Tumor Infiltrate	Pearson correlation	0.307
	Significance (two-tailed)	0.003**
	*N*	92
Expression of Ki67	Pearson correlation	0.255
	Significance (two-tailed)	0.014*
	*N*	93
Age	Pearson correlation	0.253
	Significance (two-tailed)	0.016*
	*N*	91

### PSMC2 promoted gastric cancer cells proliferation in vitro and tumor growth in vivo

For the purpose of investigating the role of PSMC2 in gastric cancer, three shPSMC2 lentiviruses (shPSMC2-1, shPSMC2-2 shPSMC2-3) were designed and infected in the MGC-803 cells. ShPSMC2-1 had the most obvious inhibitory effect on PSMC2 expression, was used in subsequent studies (Supplementary Fig. S1A). Fluorescence observation results showed the infection efficiency of gastric cancer cells reached more than 80% (Supplementary Fig. S1B). The results of qPCR and WB suggested that the mRNA and protein levels of PSMC2 were obviously decreased by shPSMC2 (Supplementary Fig. S1C–S1D). These results indicated that PSMC2 knockdown gastric cancer cells (AGS and MGC-803) were successfully constructed.

**Fig. 2:**
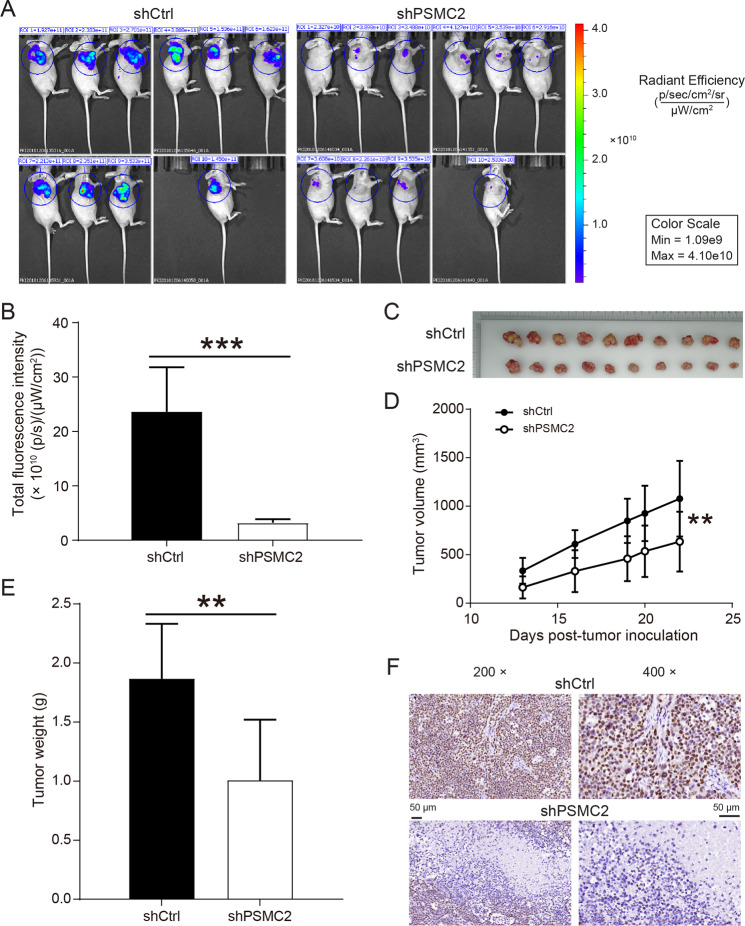
PSMC2 knockdown suppressed the growth of tumor in vivo. **A** Representative images of in vivo imaging. **B** The fluorescence intensity of nude mice in the PSMC3 knockdown group and control group was detected. **C** Pictures of solid tumors taken out of nude mice. **D** The tumor volume growth rate of nude mice in the PSMC2 knockdown group was smaller than that in the control group. **E** The tumor weight of nude mice in the PSMC2 knockdown group was lower than that in the control group. **F** The immumohistochemical staining was performed to detect the Ki67 expression in the tumor tissues. **P < 0.01; ***P < 0.001, compared with shCtrl group. Error bars meant SD.

Subsequent MTT and clone formation assay revealed that the downregulation of PSMC2 inhibited AGS and MGC-803 cells proliferation (Fig. 1C, D). The apoptosis rate of gastric cancer cells in the shPSMC2 group was apparently higher than that in the shCtrl group (Fig. 1E). ShPSMC2 declined the levels of apoptosis proteins Bax, BIM, CD40, CD40L, cytoC, IGFBP-3, IGFBP-5, p21, p27, and p53, and increased the levels of Survivin (Supplementary Fig. S2A–C). After PSMC2 knockdown, the migration ability of AGS and MGC-803 cells was also restrained (Fig. 1F). Besides, in vivo imaging experiments, the total fluorescence intensity of nude mice in the shPSMC2 group was significantly lower than that in the shCtrl group (Fig. 2A, B). Both volume and weight of tumors in the PSMC2 knockdown group were markedly smaller than those in the shCtrl group (Fig. 2C–E). The results of immunohistochemical staining indicated that was PSMC2 downregulation inhibited Ki67 expression, which further suggested that PSMC2 knockdown inhibited tumor cell proliferation (Fig. 2F).

### PSMC2 promoted the progression of gastric cancer by regulating RPS15A/mTOR pathway

**Fig. 3:**
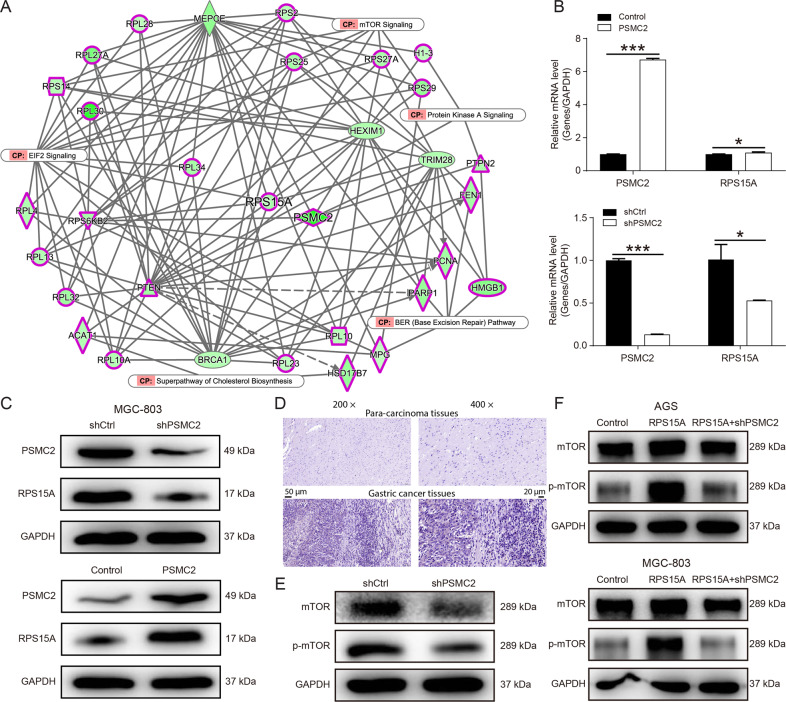
PSMC2 activated mTOR pathway by upregulating RPS15A. **A** IPA was employed to map the interaction network between molecules and pathways significantly related to PSMC2. **B** qPCR was conducted to measure the levels of PSMC2 and RPS15A mRNA in MGC-803 cells after PSMC2 overexpression or knockdown. **C** WB was performed to measure the levels of PSMC2 and RPS15A proteins in MGC-803 cells after PSMC2 overexpression or knockdown. **D** Immumohistochemical staining was conducted to evaluate the expression of RPS15A in the gastric cancer tissues and para-carcinoma tissues. **E** WB was employed to detect the expression of mTOR pathway-related proteins after PSMC2 knockdown. **F** The expression of mTOR pathway-related proteins in MGC-803 cells with RPS15A overexpression or RPS15A overexpression and PSMC2 knockdown. *P < 0.05, ***P < 0.001, compared with Control group. Error bars meant SD.

To explore the molecular mechanism of PSMC2 in gastric cancer progression, hierarchical clustering difference analysis was performed on GeneChip results of SGC-7901 cells infected with shPSMC2 lentivirus or negative control shRNA (Supplementary Fig. S3A). 20 differentially expressed genes were selected for qPCR and it was found that PSMC2 knockdown significantly inhibited RPS15A mRNA expression (Supplementary Fig. S3B). Further WB results indicated that PSMC2 knockdown markedly diminished the levels of RPS15A protein (Supplementary Fig. S3C). Besides, IPA results showed an interaction between PSMC2 and RPS15A/mTOR pathway, suggesting that PSMC2 might be involved in the progression of gastric cancer through RPS15A/mTOR pathway(Fig. 3A). Subsequent experiments revealed that PSMC2 overexpression augmented the expression of RPS15A at the transcription and translation levels, while PSMC2 knockdown diminished the expression of RPS15A (Fig. 3B, C). And RPS15A was also upregulated in gastric cancer tissues (Fig. 3D). This revealed that overexpression of PSMC2 upregulated the expression of RPS15A in gastric cancer. Furthermore, PSMC2 knockdown reduced the phosphorylation levels of mTOR protein (Fig. 3E). However, overexpression of RPS15A promoted the phosphorylation of mTOR protein and relieved the inhibition of mTOR phosphorylation by PSMC2 knockdown (Fig. 3F). These results confirmed that PSMC2 activated the mTOR pathway by upregulating RPS15A. Therefore, we further explored the effect of PSMC2/RPS15A/mTOR pathway on gastric cancer.

**Fig. 4:**
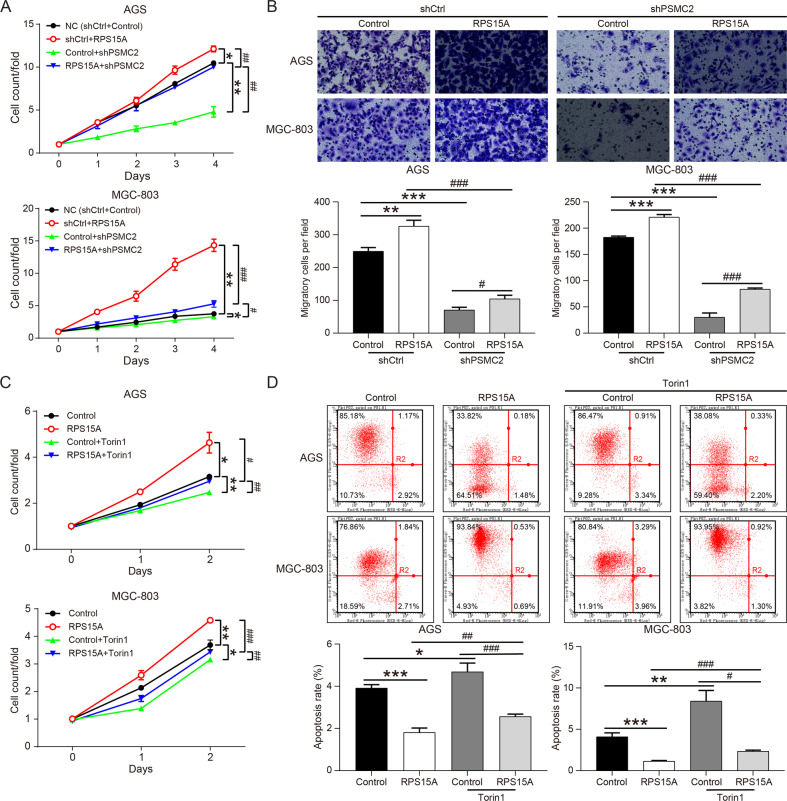
RPS15A partially restored the inhibition of gastric cancer cell proliferation induced by shPSMC2 or Torin1. **A** CCK8 assay was used to analyze the proliferation of AGS and MGC-803 cells after upregulating RPS15A or downregulating PSMC2. **B** Transwell assay were conducted to determine the changes in the migration ability of gastric cancer cells after upregulating RPS15A or downregulating PSMC2. **C** AGS and MGC-803 cells overexpressing RPS15A were treated with or without Torin1 (60 μM), and the subjected to CCK8 assay to assess the proliferation. **D** AGS and MGC-803 cells overexpressing RPS15A were treated with or without Torin1, and the subjected to flow cytometry to assess the apoptosis levels. *P < 0.05, **P < 0.01, ***P < 0.001, compared with NC or Control group. ^#^*P* < 0.05, ^##^*P* < 0.01, ^###^*P* < 0.001, compared with RPS15A overexpression or PSMC2 knockdown group or Control+Torin1 group. Error bars meant SD.

Gastric cancer cells (AGS and MGC-803 cells) with RPS15A overexpression or RPS15A overexpression and PSMC2 knockdown were constructed in vitro. The expression of RPS15A was restrained by PSMC2 knockdown, which was mitigated by overexpression of RPS15A (Supplementary Fig. S4A). Moreover, overexpression of RPS15A enhanced the proliferation and migration of AGS and MGC-803 cells, and partially alleviated the inhibition of proliferation and migration caused by PSMC2 downregulation (Fig. 4A, B). Consequently, PSMC2 promoted the proliferation and migration of gastric cancer cells by upregulating RPS15A. We attested that RPS15A overexpression increased the phosphorylation levels of mTOR protein in MGC-803 cells, but after treatment with the mTOR pathway inhibitor Torin1, the phosphorylation levels of mTOR were basically returned to the control levels (Supplementary Fig. S4B). Torin1 restricted the proliferation ability of gastric cancer cells and induced apoptosis, and also restored the accelerated proliferation and the reduced apoptosis rate caused by overexpression of RPS15A (Fig. 4C, D). In summary, PSMC2 activated mTOR pathway through upregulation of RPS15A, thus promoting the progression of gastric cancer.

### The mechanism of PSMC2 regulating RPS15A was explored

**Fig. 5:**
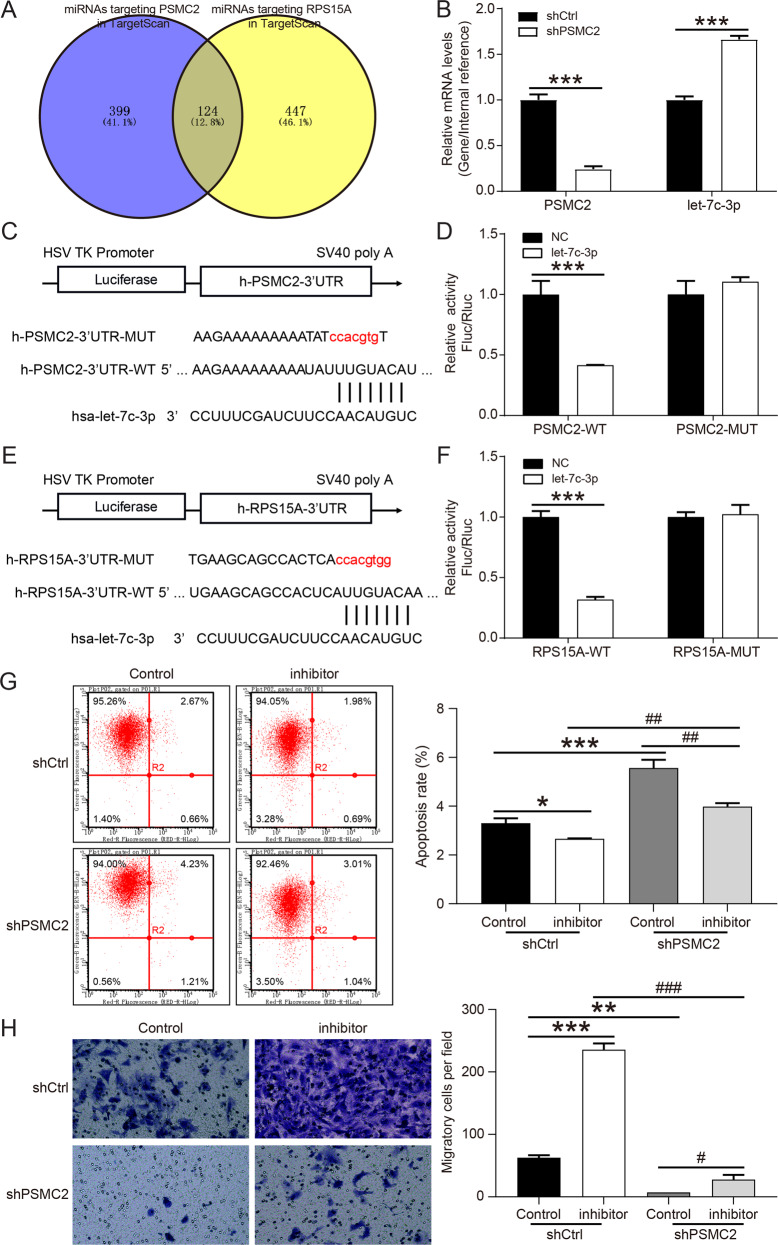
PSMC2 regulated the expression of RPS15A through competitive binding with hsa-let-7c-3p. (MGC-803). **A** Venny online tool was used to intersect the miRNAs targeted by PSMC2 and the miRNAs targeted by RPS15A. **B** The levels of PSMC2 mRNA and hsa-let-7c-3p in MGC-803 cells were determined by qPCR after PSMC2 knockdown. **C** Schematic diagram of the binding of hsa-let-7c-3p to h-PSMC2-3’UTR target site, and the mutation sequence of the PSMC2 gene. **D** The dual-luciferase reporter assay was conducted to detect the interaction between hsa-let-7c-3p and PSMC2. **E** Schematic diagram of the binding of hsa-let-7c-3p to h-RPS15A-3’UTR target site, and the mutation sequence of the RPS15A gene. **F** The dual-luciferase reporter assay was performed to detect the interaction between hsa-let-7c-3p and RPS15A. **G** The apoptosis of MGC-803 cells after inhibiting hsa-let-7c-3p or downregulating PSMC2 was determined by flow cytometry. **H** Transwell assay were conducted to determine the changes in the migration ability of MGC-803 cells after inhibiting hsa-let-7c-3p or downregulating PSMC2. *P < 0.05, **P < 0.01, ***P < 0.001, compared with shCtrl+Control group. ^#^*P* < 0.05, ^##^*P* < 0.01, ^###^*P* < 0.001, compared with hsa-let-7c-3p inhibitor or PSMC2 knockdown group. Error bars meant SD.

In view of the fact that miRNAs performed post-transcriptional manipulation on mRNAs to promote mRNA degradation and/or translation inhibition [15], we predicted miRNAs targeting PSMC2 and RPS15A in TargetScan database and found 124 miRNAs targeting both PSMC2 and RPS15A (Fig. 5A). Through the analysis of gastric cancer related data in TCGA database, tit was found that only 7 miRNAs were downregulated in gastric cancer, and 4 miRNAs with log_2_FC < −1.0 (hsa-miR-551b-5p, hsa-miR-5680, hsa-miR-4732-3p and hsa-let-7c-3p) were selected for further verification (Supplementary Table 4). We first detected the expression of miRNAs in MGC-803 cells after PSMC2 knockdown, and found that hsa-let-7c-3p was significantly upregulated (Fig. 5B). Subsequently, the results of dual-luciferase reporter assay showed that hsa-let-7c-3p not only targeted PSMC2 (Fig. 5C, D), but also targeted RPS15A (Fig. 5E, F). When the expression of hsa-let-7c-3p was inhibited, the apoptosis of MGC-803 cells was restricted, while the migration was augmented (Fig. 5G, H). PSMC2 knockdown partially alleviated the restriction of hsa-let-7c-3p inhibitor on MGC-803 cell apoptosis and the promotion of migration (Fig. 5G, H). These results determined that PSMC2 upregulated RPS15A expression by targeting hsa-let-7c-3p, thereby promoting the progression of gastric cancer.

## Discussion

As one of the most common malignant tumors in the world, gastric cancer is characterized by high morbidity, high metastasis rate, high mortality, low radical resection rate, and poor prognosis. The 5-year overall survival rate for patients with advanced gastric cancer is only 5% [16, 17]. Therefore, it is of great significance to find new therapeutic target and more effective treatment methods for gastric cancer. In this study, the levels of PSMC2 in gastric cancer tissues were significantly higher than that in para-carcinoma tissues, and the expression of PSMC2 was positively correlated with pathological stage, Tumor Infiltrate, and Ki67 expression. PSMC2, located in the genome 7q22.1 – q22.3, was highly expressed in colorectal cancer, suggesting a poor prognosis [18]. Besides, PSMC2 facilitated the proliferation and migration of osteosarcoma cells [19], and PSMC2 knockdown inhibited pancreatic cancer cell proliferation and induced apoptosis [20]. Liu *et al*. indicated that knockout of PSMC2 inhibited the proliferation of liver cancer cells and suppressed the tumorigenesis in vivo [21]. Our study obtained similar results, suggesting that the high expression of PSMC2 was associated with the low overall survival of gastric cancer patients. Further cell function tests revealed that downregulation of PSMC2 inhibited the proliferation, clone formation and migration of gastric cancer cells, and enhanced apoptosis. According to the results of Human Apoptosis Antibody Array, PSMC2 knockdown increased the levels of apoptosis-related proteins Bax, BIM, CD40, CD40L, cytoC, IGFBP-3, IGFBP-5, p21, p27, and p53 in gastric cancer cells, revealing that PSMC2 might induce cell apoptosis by regulating these apoptosis-related proteins. Furthermore, downregulation of PSMC2 decreased the levels of Ki67 and suppressed tumor growth. Hence, PSMC2 downregulation inhibited the progression of gastric cancer.

PSMC2 also played a role in tumor progression by regulating the expression of some tumor-related genes. Through bioinformatic analysis of the sequencing results of PSMC2 knockdown gastric cancer cells, it was found that PSMC2 overexpression upregulated ribosomal protein S15A (RPS15A) at the transcription and translation levels. Besides, PSMC2 knockdown inhibited the phosphorylation of mTOR, and RPS15A overexpression restored the inhibitory effect of PSMC2 knockdown on mTOR pathway. These results demonstrated that PSMC2 activated mTOR pathway through upregulation RPS15A. RPS15A located on human chromosome 16p12.3 promoted the binding of mRNA and ribosome in the early stage of translation progression, and the abnormal expression of RPS15A played a crucial role in development and progression of tumor [22, 23]. Previous studies indicated that the overexpression of RPS15A in liver cancer was associated with poor overall survival and promoted the angiogenesis of liver cancer [24]. RPS15A was upregulated in pancreatic cancer and breast cancer, and RPS15A knockdown inhibited cancer cell proliferation and induced apoptosis [25, 26]. In this study, RPS15A was also significantly upregulated in gastric cancer. Knockdown of RPS15A decreased the proliferation and migration ability of gastric cancer cells, whereas overexpression of RPS15A alleviated the inhibition of PSMC2 knockdown on the proliferation and migration. These results were similar to the study of Liu et al., determining that RPS15A was upregulated in gastric cancer, and promoted the malignant progression of gastric cancer cells [27]. However, the role and mechanism of PSMC2 regulating mTOR pathway through RPS15A in gastric cancer was not reported. There was evidence that mTOR pathway was involved in malignant progression and resistance to treatment through the excessive activation of several mechanisms [28]. Yan Li *et al*. found that S100A10 promoted glycolysis and proliferation by activing the mTOR pathway [29]. Besides, several inhibitors of the PI3K/Akt/mTOR signaling pathway were successfully developed in drug research and their efficacy was tested in gastric cancer with significant improvement [30]. To verify whether PSMC2 promoted gastric cancer progression through RPS15A/mTOR pathway, RPS15A overexpression gastric cancer cells were treated with Torin1, and the results indicated that Torin1 partially restored the proliferation promotion and apoptosis inhibition of RPS15A overexpression on gastric cancer. However, the specific mechanism by which PSMC2 regulated the expression of RPS15A was still unclear.

More and more studies showed that miRNAs were connected to the 3’UTR of mRNA through base complementary pairing for post-transcriptional operations to promote mRNA degradation and/or translation inhibition, thereby inhibiting gene expression [31, 32]. We used TargetScan and TCGA databases to predict 7 downregulated miRNAs in gastric cancer that not only bound to PSMC2 but also targeted RPS15A. Cell experiments demonstrated that hsa-let-7c-3p targeted both PSMC2 and RPS15A, and PSMC2 knockdown markedly upregulated the levels of hsa-let-7c-3p in MGC-803 cells. These results implied that PSMC2 upregulated RPS15A by competitively binding hsa-let-7c-3p. It was been demonstrated that miR-let-7c inhibited cell growth and metastasis, thus suppressing the recurrence of mucosal melanoma [33]. Moreover, hsa-let-7c-3p restrained the apoptosis and autophagy of lens epithelial cells induced by oxidative stress [34]. Our results manifested hsa-let-7c-3p also had antitumor effects in gastric cancer. Inhibition of hsa-let-7c-3p expression reduced cell apoptosis and promoted the migration of MGC-803 cells, which were reversed by PSMC2 knockdown.

In summary, both PSMC2 and RPS15A were significantly overexpressed in gastric cancer, and PSMC2 upregulated RPS15A by inhibiting the expression of hsa-let-7c-3p. In addition, we also confirmed that the promoting effect of PSMC2/RPS15A on gastric cancer progression, suggesting that PSMC2/RPS15A may be potential targets for targeted therapy of gastric cancer. However, there are still some deficiencies in this study, that is, more clinical samples are needed to detect the expression of hsa-let-7c-3p in gastric cancer, and to verify the correlation between PSMC2, hsa-let-7c-3p and RPS15A.

## Supplementary information

Supplementary tables

Supplementary figures

## Data Availability

The data used and analyzed during the current study are available from the corresponding author on reasonable request.

## References

[1] Sung H, Ferlay J, Siegel RL, Laversanne M, Soerjomataram I, Jemal A (2021). Global cancer statistics 2020: GLOBOCAN estimates of incidence and mortality worldwide for 36 cancers in 185 countries. CA Cancer J Clin..

[2] Smyth EC, Nilsson M, Grabsch HI, van Grieken NC, Lordick F (2020). Gastric cancer. Lancet.

[3] Russo AE, Strong VE (2019). Gastric cancer etiology and management in Asia and the West. Annu Rev Med.

[4] Fu M, Gu J, Jiang P, Qian H, Xu W, Zhang X (2019). Exosomes in gastric cancer: roles, mechanisms, and applications. Mol Cancer.

[5] Yuan L, Xu ZY, Ruan SM, Mo S, Qin JJ, Cheng XD (2020). Long non-coding RNAs towards precision medicine in gastric cancer: early diagnosis, treatment, and drug resistance. Mol Cancer.

[6] Dong Y, Zhang S, Wu Z, Li X, Wang WL, Zhu Y (2019). Cryo-EM structures and dynamics of substrate-engaged human 26S proteasome. Nature.

[7] Finley D (2009). Recognition and processing of ubiquitin-protein conjugates by the proteasome. Annu Rev Biochem.

[8] Smith DM, Fraga H, Reis C, Kafri G, Goldberg AL (2011). ATP binds to proteasomal ATPases in pairs with distinct functional effects, implying an ordered reaction cycle. Cell..

[9] Kaneko T, Hamazaki J, Iemura S, Sasaki K, Furuyama K, Natsume T (2009). Assembly pathway of the Mammalian proteasome base subcomplex is mediated by multiple specific chaperones. Cell..

[10] Nijhawan D, Zack TI, Ren Y, Strickland MR, Lamothe R, Schumacher SE (2012). Cancer vulnerabilities unveiled by genomic loss. Cell..

[11] Zhou B, Peng K, Wang G, Chen W, Kang Y (2021). Silencing proteasome 26S subunit ATPase 2 (PSMC2) protects the osteogenic differentiation in vitro and osteogenesis in vivo. Calcif Tissue Int.

[12] Song M, Wang Y, Zhang Z, Wang S (2017). PSMC2 is up-regulated in osteosarcoma and regulates osteosarcoma cell proliferation, apoptosis and migration. Oncotarget..

[13] Su Y, Zeng Z, Rong D, Yang Y, Wu B, Cao Y (2021). PSMC2, ORC5 and KRTDAP are specific biomarkers for HPV-negative head and neck squamous cell carcinoma. Oncol Lett.

[14] Guan Y, Xu F, Wang Y, Tian J, Wan Z, Wang Z (2020). Identification of key genes and functions of circulating tumor cells in multiple cancers through bioinformatic analysis. BMC Med Genomics.

[15] Mulholland EJ, Green WP, Buckley NE, McCarthy HO (2019). Exploring the potential of MicroRNA Let-7c as a therapeutic for prostate cancer. Mol Ther Nucleic Acids.

[16] Gambardella V, Castillo J, Tarazona N, Gimeno-Valiente F, Martinez-Ciarpaglini C, Cabeza-Segura M (2020). The role of tumor-associated macrophages in gastric cancer development and their potential as a therapeutic target. Cancer Treat Rev.

[17] Shafabakhsh R, Yousefi B, Asemi Z, Nikfar B, Mansournia MA, Hallajzadeh J (2020). Chitosan: a compound for drug delivery system in gastric cancer-a review. Carbohydr Polym.

[18] He J, Xing J, Yang X, Zhang C, Zhang Y, Wang H (2019). Silencing of proteasome 26S subunit ATPase 2 regulates colorectal cancer cell proliferation, apoptosis, and migration. Chemotherapy..

[19] Li GW, Yan X (2019). Lower miR-630 expression predicts poor prognosis of osteosarcoma and promotes cell proliferation, migration and invasion by targeting PSMC2. Eur Rev Med Pharm Sci.

[20] Qin J, Wang W, An F, Huang W, Ding J (2019). PSMC2 is up-regulated in pancreatic cancer and promotes cancer cell proliferation and inhibits apoptosis. J Cancer.

[21] Liu Y, Chen H, Li X, Zhang F, Kong L, Wang X (2021). PSMC2 regulates cell cycle progression through the p21/Cyclin D1 pathway and predicts a poor prognosis in human hepatocellular carcinoma. Front Oncol.

[22] Wool IG, Chan YL, Gluck A (1995). Structure and evolution of mammalian ribosomal proteins. Biochem Cell Biol.

[23] Linder P, Prat A (1990). Baker’s yeast, the new work horse in protein synthesis studies: analyzing eukaryotic translation initiation. Bioessays..

[24] Guo P, Wang Y, Dai C, Tao C, Wu F, Xie X (2018). Ribosomal protein S15a promotes tumor angiogenesis via enhancing Wnt/beta-catenin-induced FGF18 expression in hepatocellular carcinoma. Oncogene..

[25] Liang J, Liu Y, Zhang L, Tan J, Li E, Li F (2019). Overexpression of microRNA-519d-3p suppressed the growth of pancreatic cancer cells by inhibiting ribosomal protein S15A-mediated Wnt/beta-catenin signaling. Chem Biol Interact.

[26] Kong L, Wei Q, Hu X, Chen L, Li J (2020). Ribosomal protein small subunit 15A (RPS15A) inhibits the apoptosis of breast cancer MDA-MB-231 cells via upregulating phosphorylated ERK1/2, Bad, and Chk1. J Cell Biochem.

[27] Liu C, He X, Liu X, Yu J, Zhang M, Yu F (2019). RPS15A promotes gastric cancer progression via activation of the Akt/IKK-beta/NF-kappaB signalling pathway. J Cell Mol Med.

[28] Zanini S, Renzi S, Giovinazzo F, Bermano G (2020). mTOR pathway in gastroenteropancreatic neuroendocrine tumor (GEP-NETs). Front Endocrinol.

[29] Li Y, Li XY, Li LX, Zhou RC, Sikong Y, Gu X (2020). S100A10 accelerates aerobic glycolysis and malignant growth by activating mTOR-signaling pathway in gastric cancer. Front Cell Dev Biol.

[30] Baghery Saghchy Khorasani A, Pourbagheri-Sigaroodi A, Pirsalehi A, Safaroghli-Azar A, Zali MR, Bashash D (2021). The PI3K/Akt/mTOR signaling pathway in gastric cancer; from oncogenic variations to the possibilities for pharmacologic interventions. Eur J Pharm.

[31] Ayub SG, Kaul D, Ayub T (2015). Microdissecting the role of microRNAs in the pathogenesis of prostate cancer. Cancer Genet.

[32] Gu S, Rong H, Zhang G, Kang L, Yang M, Guan H (2016). Functional SNP in 3’-UTR microRNA-binding site of ZNF350 confers risk for age-related cataract. Hum Mutat.

[33] Tang H, Ma M, Dai J, Cui C, Si L, Sheng X (2019). miR-let-7b and miR-let-7c suppress tumourigenesis of human mucosal melanoma and enhance the sensitivity to chemotherapy. J Exp Clin Cancer Res.

[34] Li T, Huang Y, Zhou W, Yan Q (2020). Let-7c-3p regulates autophagy under oxidative stress by targeting ATG3 in lens epithelial cells. Biomed Res Int.

