# Supplementary material for: PSMC2 promotes the progression of gastric cancer via induction of RPS15A/mTOR pathway
Source: Oncogenesis. 2022 Mar 7;11(1):12. doi: 10.1038/s41389-022-00386-7 (PMC8901802; doi:10.1038/s41389-022-00386-7)
Supplement: Supplementary file 1 — Supplementary tables [file 41389_2022_386_MOESM1_ESM.docx]

**Supplementary table 1.** Antibodies used in western blotting, IHC and Co-IP

| Primary antibodies | Dilution in WB/Co-IP | Source species | Company | Catalog No. |
| --- | --- | --- | --- | --- |
| PSMC2 | 1:1000 | Mouse | Santa Cruz | SC-166972 |
| CDK1 | 1:1000 | Rabbit | abcam | Ab133327 |
| FAM111B | 1:1000 | Rabbit | novus | NBP1-86645 |
| LMNB1 | 1:1000 | Rabbit | abcam | Ab229025 |
| RPS15A | 1:750 | Rabbit | Invitrogen | PA5-51314 |
| mTOR | 1:3000 | Mouse | Proteintech | 66888-1-lg |
| p-mTOR | 1:3000 | Mouse | Proteintech | 67778-1-lg |
| GAPDH | 1:3000 | Rabbit | Bioworld | AP0063 |
| Primary antibodies | Dilution in IHC | Source species | Company | Catalog No. |
| PSMC2 | 1:200 | Mouse | Santa Cruz | SC-166972M |
| Ki67 | 1:200 | Rabbit | abcam | Ab16667 |
| RPS15A | 1:200 | Rabbit | Invitrogen | PA5-51314 |
| Secondary antibody | Dilution |  | Company | Catalog No. |
| HRP Goat Anti-Mouse IgG (WB and Co-IP) | 1:3000 |  | Beyotime | A0216 |
| HRP Goat Anti-Rabbit IgG (WB and Co-IP) | 1:3000 |  | Beyotime | A0208 |
| HRP Goat Anti-Rabbit IgG (IHC) | 1:400 |  | Abcam | Ab6721 |
| HRP Goat Anti-Mouse IgG (IHC) | 1:400 |  | Beyotime | A0216 |

**Supplementary table 2.** Target sequences and shRNA sequences used for gene knockdown

| Gene symbol | Target sequence |  | shRNA sequences (5'-3') |
| --- | --- | --- | --- |
| PSMC2-1 | GCCAGGGAGATTGGATAGAAA | Pbr00145-a | ccggGCCAGGGAGATTGGATAGAAAttcaagagaTTTCTATCCAATCTCCCTGGCTTTTTg |
|  |  | Pbr00145-b | aattcaaaaaGCCAGGGAGATTGGATAGAAAttcaagagaTTTCTATCCAATCTCCCTGGC |
| PSMC2-2 | CAACGTAAAGCAGTTTGCCAA | Pbr23881-a | ccggCAACGTAAAGCAGTTTGCCAActcgagTTGGCAAACTGCTTTACGTTGTTTTTg |
|  |  | Pbr23881-b | aattcaaaaaCAACGTAAAGCAGTTTGCCAActcgagTTGGCAAACTGCTTTACGTTG |
| PSMC2-3 | AAGCAAGTTGAAGATGACATT | Pbr23882-a | ccggAAGCAAGTTGAAGATGACATTctcgagAATGTCATCTTCAACTTGCTTTTTTTg |
|  |  | Pbr23882-b | aattcaaaaaAAGCAAGTTGAAGATGACATTctcgagAATGTCATCTTCAACTTGCTT |
| RPS15A-1 | GTGCAACTCAAAGACCTGGAA | Pbr00142-a | ccggGTGCAACTCAAAGACCTGGAActcgagTTCCAGGTCTTTGAGTTGCACTTTTTg |
|  |  | Pbr00142-b | aattcaaaaaGTGCAACTCAAAGACCTGGAActcgagTTCCAGGTCTTTGAGTTGCAC |
| RPS15A-2 | GTGCAACTCAAAGACCTGGAA | Pbr00143-a | ccggGTGCAACTCAAAGACCTGGAActcgagTCTGCCAGGACATTCATGCGCTTTTTg |
|  |  | Pbr00143-b | aattcaaaaaGTGCAACTCAAAGACCTGGAActcgagTCTGCCAGGACATTCATGCGC |
| RPS15A-3 | GATGACCACAGAGCTGGGAAA | Pbr00144-a | ccggGATGACCACAGAGCTGGGAAActcgagTTTCCCAGCTCTGTGGTCATCTTTTTg |
|  |  | Pbr00144-b | aattcaaaaaGATGACCACAGAGCTGGGAAActcgagTTTCCCAGCTCTGTGGTCATC |

**Supplementary table 3.** Primers used in qPCR

| Gene | Forward primer sequence (5'-3') | Reverse primer sequence (5'-3') |
| --- | --- | --- |
| PSMC2 | CAGCACTCTGGGATTTGGCT | TTTCTATCCACGCCCACTCTC |
| CCNE2 | AGCTGGTCTGGCGAGGTTTT | GGCCTGGATTATCTGGGCTTC |
| CDK1 | CCATACCCATTGACTAACTAT | ACCCCTTCCTCTTCACTTTC |
| E2F1 | CACTTTCGGCCCTTTTGCTC | GTGCTCTCACCGTCCTACAC |
| ID3 | GAGCTTGCTGGACGACATGA | TGTAGGATTTCCACCTGGCTAA |
| ID2 | CCGTGAGGTCCGTTAGGAAA | TGAGCTTGGAGTAGCAGTCG |
| PRSS23 | GAAGGAAGCGGCAGATTTATGG | TGATGTTGAGAAAGGGTAGTTGAGC |
| FAM111B | CCAGACAATTCCCAGGATTAGA | TAGCATACCGCCTACCCAGA |
| IFITM1 | CGTGAAGTCTAGGGACAGGAAG | GCCGAATACCAGTAACAGGATGA |
| BAMBI | TCATTGCCGTGCCCATTGCT | ATCTGTTGCCGCTGATCCTG |
| PBX3 | TCCGAGAACAGAGTAGAACACG | GAGCTGCATCTGAATGGAACTAA |
| SOX4 | CTCAAAGACAGCGACAAGATCC | ACTTGTAGTCGGGGTAGTCAGC |
| LMNB1 | CCCAGTTGGAAGCCTCCTTA | GCGAAACTCCAAGTCCTCAG |
| DIXDC1 | CGGTAACCAACAGGAGATGAAGA | CCGAAGTCTGGTGCATACGA |
| NUCKS1 | AAAATGTGCGCCAACAACGG | AATGGTGCCTCATCCTCCTC |
| KIAA0101 | GGATAGTTTTCGGGTCCTTGT | AGCAGCCACCACTTTTCTGTA |
| NME4 | AAGCCCTTCTACCCTGCCCTCA | GGACGACATTGTACCCTTCCCAGA |
| RPS15A | CGCGCCGCCACAATG | CACAGTGAGAAACCGGACGA |
| CENPH | AGCTTGCATTAGACAGGATGAGAC | CCCAAGATTCCTGCTGTGATT |
| U6 | CTCGCTTCGGCAGCACA | AACGCTTCACGAATTTGCGT |
| let-7c-3p | CATGGGTGCTGTACAACCTTCTAG | GTGCAGGGTCCGAGGT |
| GAPDH | TGACTTCAACAGCGACACCCA | CACCCTGTTGCTGTAGCCAAA |

**Supplementary table 4.** The expression of miRNAs targeting PSMC2 and RPS15A at the same time in gastric cancer was predicted in the TCGA database, and only the prediction results of downregulated miRNAs were shown here.

| miRNA | miRNA_Name | log_2_FoldChange | P value | Adj.P |
| --- | --- | --- | --- | --- |
| hsa-let-7c | hsa-let-7c-3p | -1.1187 | 1.84E-06 | 7.96E-06 |
| hsa-miR-379 | hsa-miR-379-5p | -0.4898 | 5.54E-04 | 1.72E-03 |
| hsa-miR-4732 | hsa-miR-4732-3p | -1.4041 | 3.32E-05 | 1.23E-04 |
| hsa-miR-4786 | hsa-miR-4786-3p | -0.8950 | 3.84E-05 | 1.41E-04 |
| hsa-miR-551b | hsa-miR-551b-5p | -2.1095 | 3.11E-16 | 5.63E-15 |
| hsa-miR-5680 | hsa-miR-5680 | -1.7731 | 3.48E-08 | 2.06E-07 |
| hsa-miR-664a | hsa-miR-664a-5p | -0.4999 | 5.40E-06 | 2.16E-05 |
