# Supplementary material for: PSMC2 promotes the progression of gastric cancer via induction of RPS15A/mTOR pathway
Source: Oncogenesis. 2022 Mar 7;11(1):12. doi: 10.1038/s41389-022-00386-7 (PMC8901802; doi:10.1038/s41389-022-00386-7)
Supplement: Supplementary file 2 — Supplementary figures [file 41389_2022_386_MOESM2_ESM.docx]

**
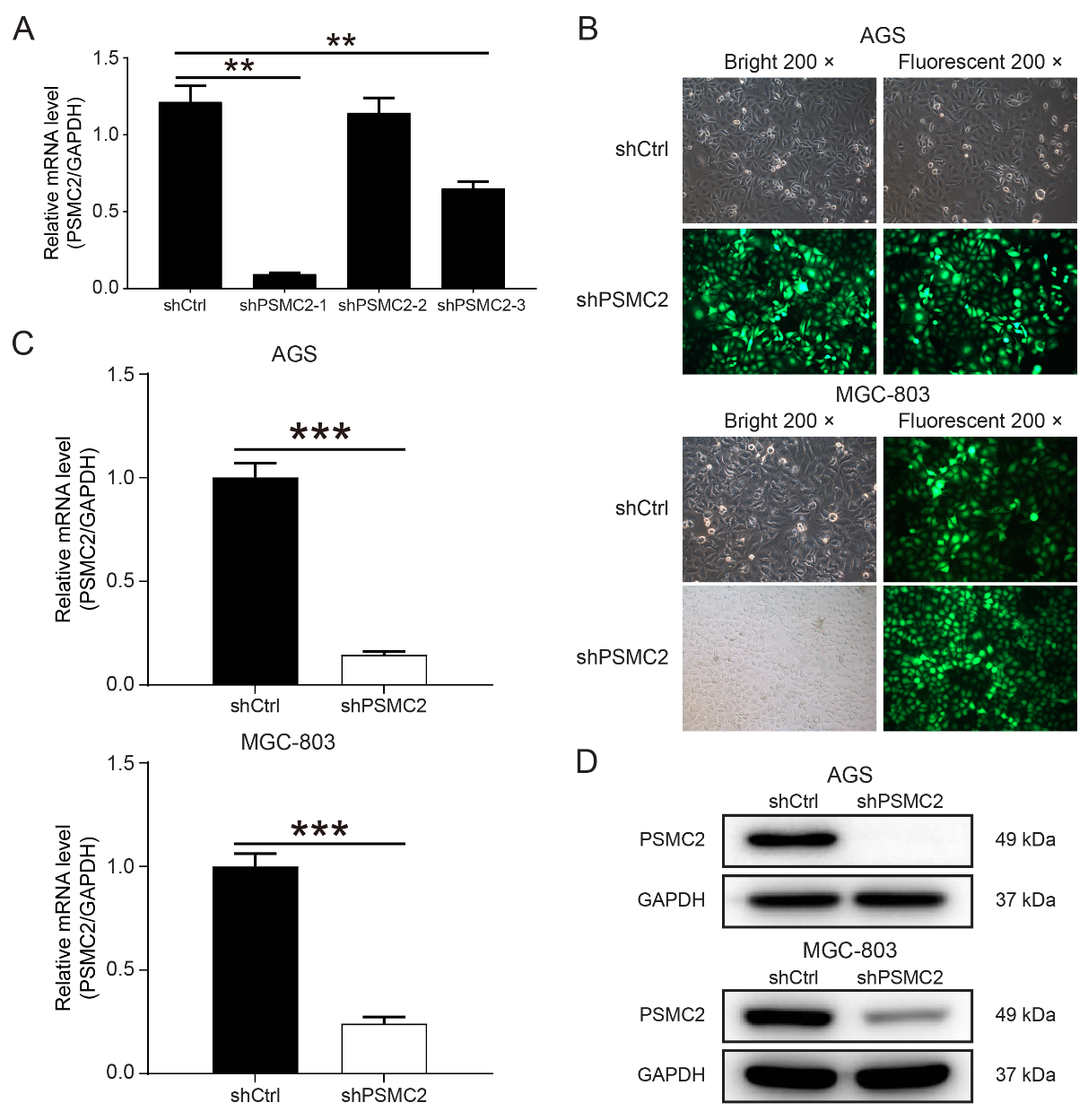
**

**Supplementary figure 1 PSMC2 knockdown gastric cancer cells were constricted successfully.**

(A) 3 interfering sequences were designed using PSMC2 gene as a template and infected gastric cancer cells. qPCR was used to detect the PSMC2 mRNA levels to evaluate the knockdown efficiency. (B) The fluorescence of GFP was observed under a microscope to assess the infection efficiency. (C) The PSMC2 mRNA levels in gastric cancer cell infected with shPSMC2 lentivirus were determined by qPCR. (D) The PSMC2 protein levels in gastric cancer cells infected with shPSMC2 lentivirus were detected using WB. ** *P* < 0.01; *** *P* < 0.001, compared with shCtrl group. Error bars meant SD.


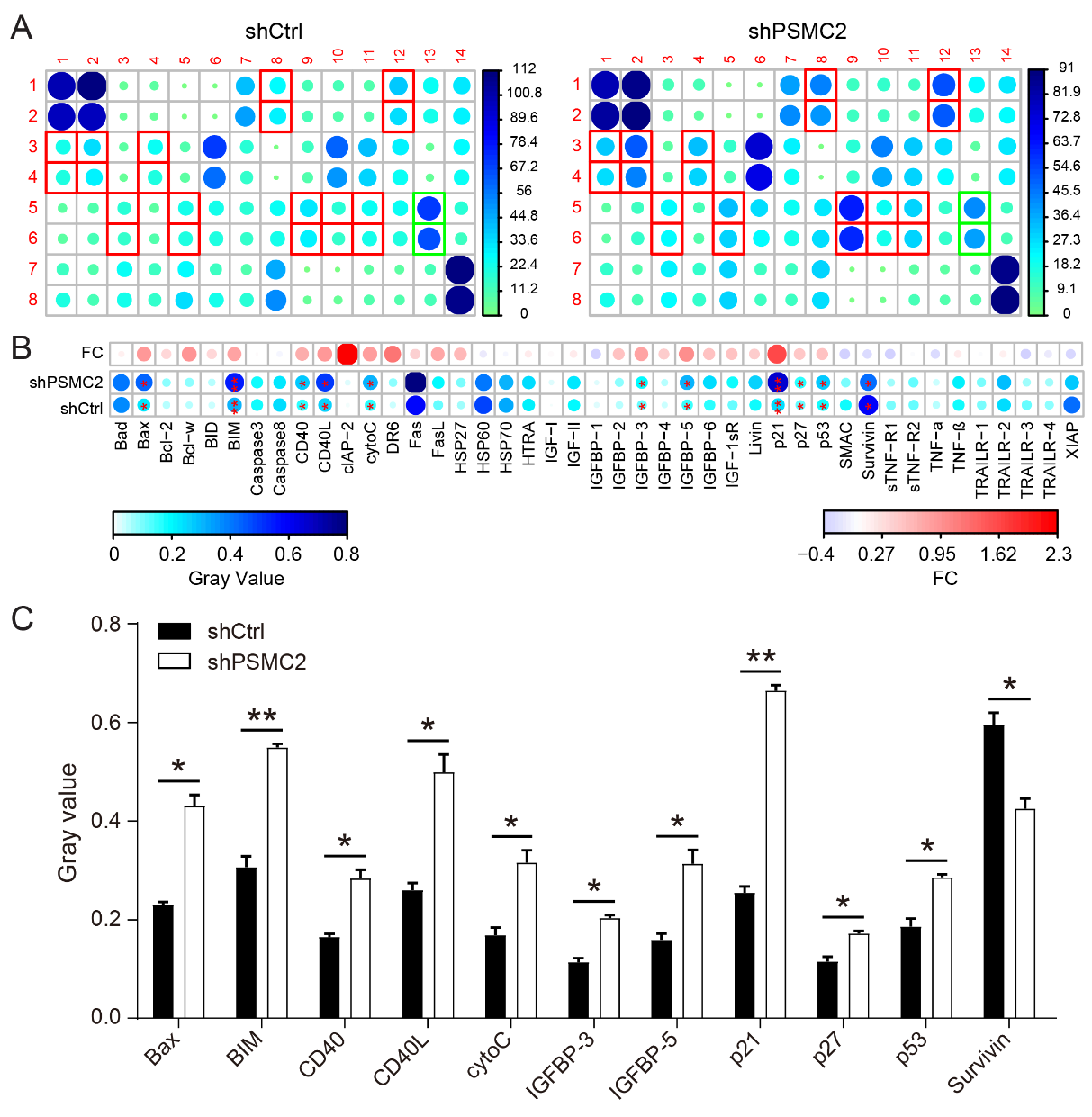


**Supplementary figure 2 The results of Human Apoptosis Antibody Array.**

(A) Distribution and detection of Human Apoptosis Antibody Array. The red box represents a significant upregulation of the protein; the green box represents a significant downregulation of the protein. (B) Comparative analysis of the detection results of Human Apoptosis Antibody Array. (C) The gray value of the proteins with significant changes in expression were plotted into a histogram and subjected statistical analysis. * *P* < 0.05; ** *P* < 0.01, compared with shCtrl group. Error bars meant SD.


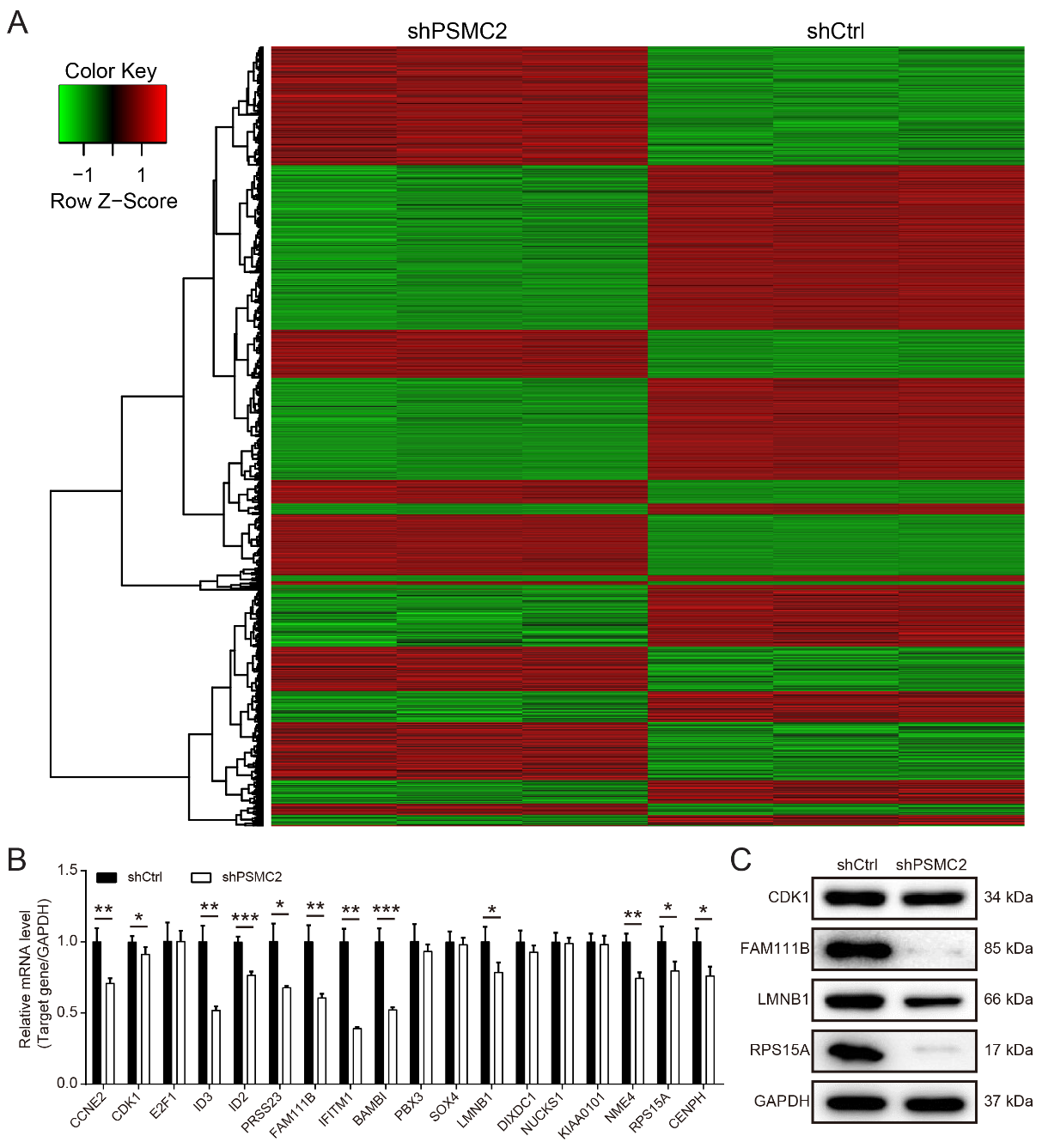


**Supplementary figure 3 The downstream gene regulated by PSMC2 was screened.**

(A) The differentially expressed genes in PSMC2 knockdown SGC-7901 cells were screened by hierarchical cluster analysis, which were displayed in the heat map. (B) Twenty differentially expressed genes in PSMC2 knockdown gastric cancer cells were selected and qPCR was used to verify the effects of PSMC2 knockdown on their mRNA expression. (C) The protein levels of CDK1, FAM111B, LMNB1 and RPS15A, which were significantly downregulated in PSMC2 knockdown MGC-803 cells were detected using WB. * *P* < 0.05; ** *P* < 0.01; *** *P* < 0.001, compared with shCtrl group. Error bars meant SD.


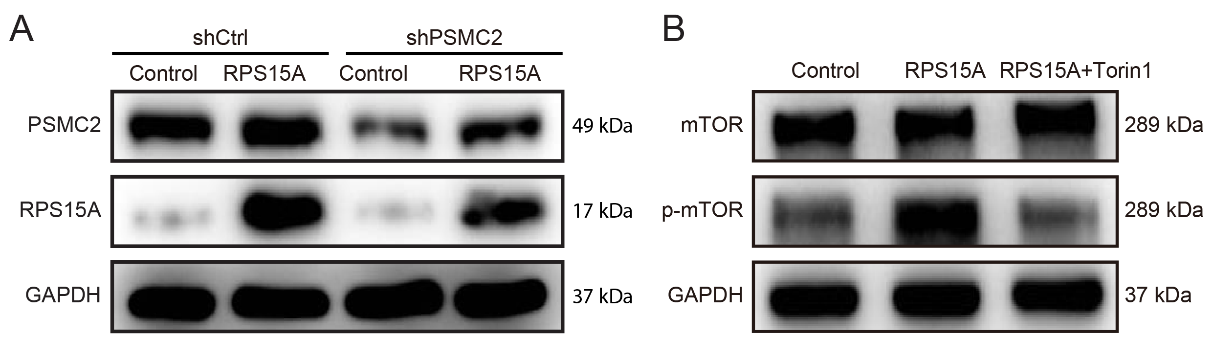


**Supplementary figure 4 RPS15A partially restored the inhibition of shPSMC2 or Torin1 on RPS15A or mTOR pathway**.

(A) WB was performed to detect the PSMC2 and RPS15A protein levels in gastric cancer cells (MGC-803) infected with RPS15A overexpression lentivirus or shPSMC2 lentivirus or both RPS15A overexpression and shPSMC2 lentivirus. (B) The expression of mTOR pathway-related proteins in RPS15A overexpression MGC-803 cells treat with or without mTOR pathway inhibitor Torin1 (60 μM).
